# Continuity vs. the Crowd—Tradeoffs Between Continuous and Intermittent Citizen Hydrology Streamflow Observations

**DOI:** 10.1007/s00267-017-0872-x

**Published:** 2017-04-25

**Authors:** Jeffrey C. Davids, Nick van de Giesen, Martine Rutten

**Affiliations:** 1 Delft University of Technology, TU Delft Building 23, Stevinweg 1, Delft, 2628 CN Netherlands; 2 SmartPhones4Water, 3881 Benatar Way, Suite G, Chico, CA 95928 USA

**Keywords:** SmartPhones4Water, Citizen science, Citizen hydrology, Subsampling, Streamflow, Nepal

## Abstract

Hydrologic data has traditionally been collected with permanent installations of sophisticated and accurate but expensive monitoring equipment at limited numbers of sites. Consequently, observation frequency and costs are high, but spatial coverage of the data is limited. Citizen Hydrology can possibly overcome these challenges by leveraging easily scaled mobile technology and local residents to collect hydrologic data at many sites. However, understanding of how decreased observational frequency impacts the accuracy of key streamflow statistics such as minimum flow, maximum flow, and runoff is limited. To evaluate this impact, we randomly selected 50 active United States Geological Survey streamflow gauges in California. We used 7 years of historical 15-min flow data from 2008 to 2014 to develop minimum flow, maximum flow, and runoff values for each gauge. To mimic lower frequency Citizen Hydrology observations, we developed a bootstrap randomized subsampling with replacement procedure. We calculated the same statistics, and their respective distributions, from 50 subsample iterations with four different subsampling frequencies ranging from daily to monthly. Minimum flows were estimated within 10% for half of the subsample iterations at 39 (daily) and 23 (monthly) of the 50 sites. However, maximum flows were estimated within 10% at only 7 (daily) and 0 (monthly) sites. Runoff volumes were estimated within 10% for half of the iterations at 44 (daily) and 12 (monthly) sites. Watershed flashiness most strongly impacted accuracy of minimum flow, maximum flow, and runoff estimates from subsampled data. Depending on the questions being asked, lower frequency Citizen Hydrology observations can provide useful hydrologic information.

## Background and Introduction

Natural resource managers rely on timely and accurate data to make management decisions. Though water resources for human purposes is one of the most fundamental ecosystem services (Buytaert et al. 2014), fundamental data required to adequately manage water resources is often lacking both spatially and temporally (Gleick 1998; Hannah et al. 2011; Shrestha et al. 2012; and others). Remarkably, despite the multiple benefits of long term hydrologic records, the amount of river flow data being collected is actually declining in many parts of the world, especially in Africa, Latin America, Asia, and even North America (Mishra and Coulibaly 2009; Van de Giesen et al. 2014). The factors leading to this decline are diverse, but include a lack of understanding of the importance of long-term streamflow data, and persistent funding challenges (Pearson 1998). This lack of information makes it difficult to know how our water systems are changing over time and space due to natural or human activities, and to decide what management actions should be taken to either avoid or mitigate undesirable conditions in the present and future. In addition to remotely sensed stream stage and flow measurement techniques (Hirsch and Costa 2004; currently applicable to large rivers only), Citizen science appears to be a promising methodology for filling these data gaps (Sanz et al. 2014; Fienen and Lowry 2012).

Citizen Science is the process of involving citizens in the scientific process as researchers (Kruger and Shannon 2000). Citizen Science can include community based monitoring (Whitelaw et al. 2003) and/or community-based management (Keough and Blahna 2006). Citizen Science is on the rise in the USA (Whitelaw et al. 2003), Canada (Savan et al. 2003), and many other areas around the world (Sultana and Abeyasekera 2008; Nagendra et al. 2005). New developments in sensing technologies, data processing and analysis techniques, and methods of knowledge communication are opening novel opportunities for Citizen Science (Buytaert et al. 2014). In particular, recent advances in mobile technologies make smartphones a perfect tool for Citizen Science. Global Positioning Systems (GPS) and high resolution camera technology embedded in smartphones can be leveraged to collect verifiable records in the field. Cellular networks and the internet can be used to transmit collected data to a central repository.

Conventional methods for collecting hydrologic data depend on fixed deployments of advanced, highly accurate, but costly monitoring equipment installed at limited numbers of monitoring locations (Turnipseed and Sauer 2010). Therefore, observational frequency and expenses are high, but spatial extent of the resulting data is limited. Achieving adequate maintenance of sophisticated equipment can be costly (Mazzoleni et al. 2015), and in developing countries often exceeds local technical and resource capacity. Experience has shown that permanently deployed monitoring equipment is susceptible to corrosion, vandalism, and theft (van Overloop et al. 2014).

Applying Citizen Science to hydrologic data collection (i.e., Citizen Hydrology) has the potential to overcome these limitations. Fienen and Lowry (2012) demonstrated that Citizen Hydrology water level measurements using text message-based reporting can have acceptable errors. Mazzoleni et al. (2015) showed that crowdsourced streamflow observations can be integrated into hydrological models to improve flood predictions, and found the accuracy of individual measurements impacted results more than the irregularities in observation assimilation. Rather than using expensive installations at a few points, Citizen Hydrology leverages mobile technology to gather data at many sites, in a manner that is highly scalable, enabling the production of significantly more data than an individual organization possibly could (O’Grady et al. 2016). One of the tradeoffs for increased spatial resolution, however, is reduced temporal resolution.

We were interested in how decreased observation frequency associated with Citizen Hydrology observations affects the ability to accurately characterize critical streamflow metrics (e.g., runoff). Based on our review of the literature using search terms of streamflow, citizen science/hydrology, subsampling, and sample frequency, we could not identify other previous works addressing this particular theme. While Moss and Tasker (1991) used subsampling to evaluate two different hydrological network design technologies in order to maximize regional stream gauge information with limited funding and monitoring period, their subsampling was based on selecting subsets of sites and site-years of data (the entire year) to develop regressions for ungauged basins. Thoreson et al. (1999) investigated the relationship between different sampling intervals and water volume calculations, but in the context of irrigation canal systems, where flows are artificially managed to meet irrigation water requirements. One possible explanation for why this theme has not been explored is that existing literature assumes traditional streamflow monitoring approaches will be used, whereby permanent water level or water velocity sensing devices are installed and used to collect samples every 15-min (if not more frequently). Perhaps, therefore, it is often implicitly assumed that high frequency data records will be available if one is interested in monitoring streamflow.

An immediate application of this research is to inform monitoring plans for a Citizen Hydrology campaign in Nepal called SmartPhones4Water-Nepal (S4W-Nepal). The initial objective of S4W-Nepal is to further constrain the water budget in the Kathmandu Valley using underutilized sources of information, including water level and streamflow data collected by Citizen Hydrologists. At streamflow monitoring locations, low-cost staff gauges will be installed, and water level data will be collected by local residents with smartphones using an open source Android data collection platform called Open Data Kit (ODK) Collect (Anokwa et al. 2009). Within ODK Collect, each water level observation will require the Citizen Hydrologist to enter the water level reading, save the current date, time, GPS coordinates, and take a photograph of the observation. The data will be automatically transmitted to a centralized Google Cloud database via ODK Aggregate. Stage-discharge curves for the selected sites will be developed from monthly to bi-monthly observations of discharge with a SonTek FlowTracker Acoustic Doppler Velocimeter performed by local BSc and MSc science and engineering students. Additional research is underway to explore the precision and accuracy of Citizen Hydrologist water level and discharge measurements. In addition to the various technical challenges, onsite training, frequent communication, and effective incentivization must also play a central role for the campaign to be successful and sustainable.

The goal of this paper is to evaluate the impacts of decreased observational frequency, which is a primary tradeoff of Citizen Hydrology observations, on estimates of minimum flow, maximum flow, and runoff. We attempt to meet this goal by performing a subsampling analysis on 7 years of data from 50 randomly selected United States Geological Survey (USGS) stream gauges in California. The three hypotheses we further evaluate are: (1) decreased observational frequency will negatively impact accuracy of flow and runoff estimates, (2) the nature of this impact will differ depending on the parameter in question, and (3) there will be correlations between accuracy of flow and runoff estimates and latitude, watershed area, Richards-Baker Flashiness Index (R-B Index), and storage ratio (see section Correlation analysis for details). The following analysis assumed (1) subsampled water level observations were as precise and accurate as continuous USGS records and (2) an equally accurate stage-discharge curve was available for converting water levels to flows. While not addressed in this paper, these simplifying assumptions highlight two important areas where further research is required if Citizen Hydrology is to help fill the globally widening hydrologic data gap.

## Materials and Methods

### Streamflow Data

**Fig. 1:**
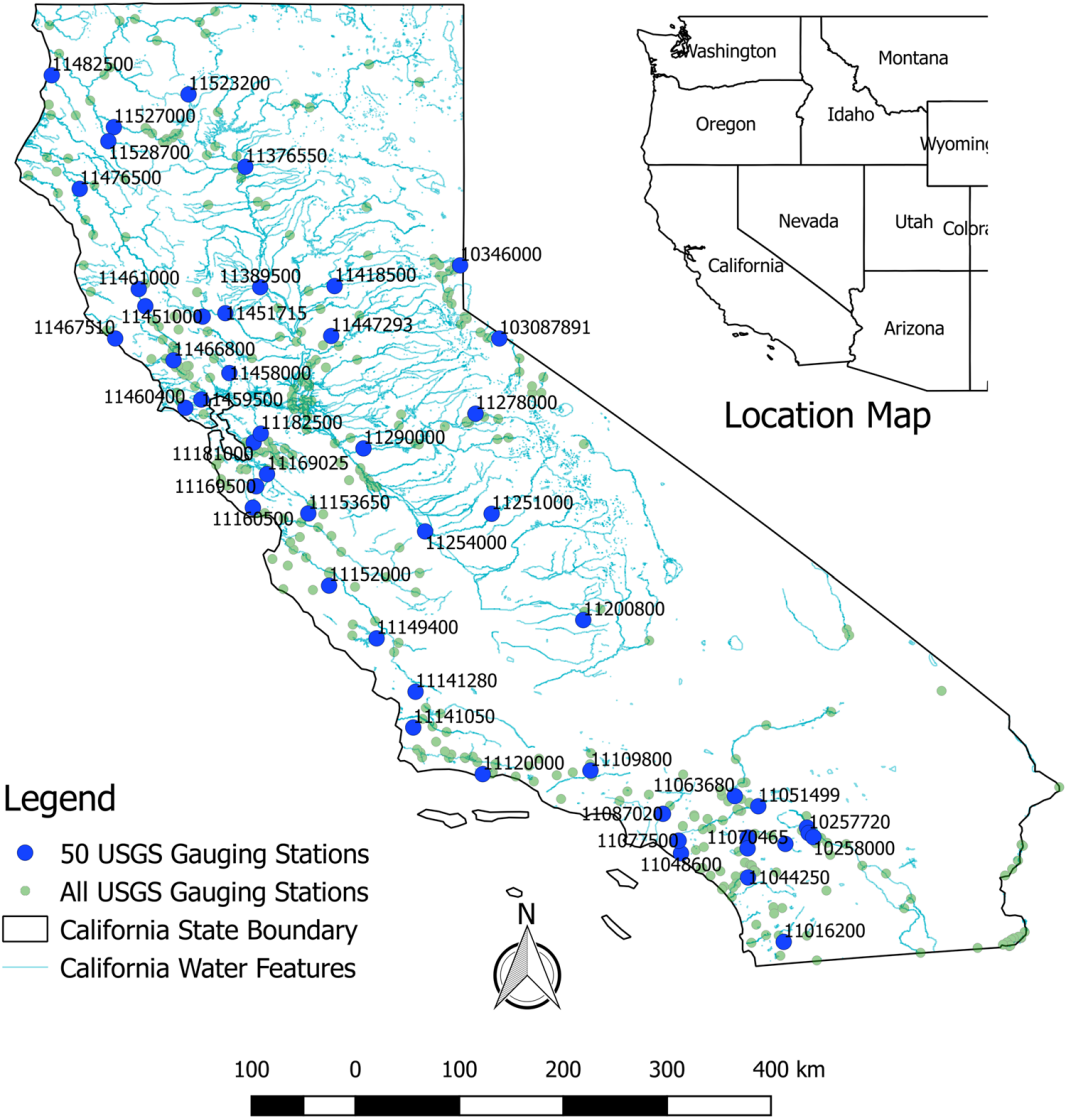
Map of California showing 50 randomly selected USGS stream gauging sites. All major waterways in the state are shown in *light blue*. A location map of the West Coast of the U.S. is also shown on the *top right*. Subsampled hydrograph results shown in section Example subsampled hydrographs are for the Truckee River Near Farad (SiteID 10346000), which is the northern most site on the California-Nevada border

We compiled an inventory of the 403 streamflow gauging stations (gauges or sites) in the state of California operated and maintained by the USGS with 15-min water level and flow data from January 1st 2008 to December 31st 2014. From this inventory, 50 streamflow gauges were randomly selected. For these 50 gauges, we compiled 15-min records and station metadata including the name, location, and elevation of the gauging station. Figure 1 shows the location of the 50 gauging stations labeled by the USGS Station ID or SiteID. Basic information about the 50 gauges is provided as supplemental material to this paper.

### Subsampling Procedure

To mimic Citizen Hydrology observations at a lower observation frequency than the continuous record, we developed a bootstrap randomized subsampling with replacement procedure to generate randomized subsample datasets from each gauge record. The subsample datasets were randomly selected from the continuous record at average subsample intervals of once a day, every three days, weekly, and monthly. The subsampling procedure was similar to that used by Jones et al. (2012) to assess the influence of sampling frequency on total phosphorus and total suspended solid loads. The subsampling algorithms detailed in Eqs 1–4 were implemented to develop multiple subsample iterations via sampling with replacement. The subsampling procedure was coded in Python (Python v2.7 2016), and is available at GitHub at https://github.com/jcdavids/CAFlowSubsample. This procedure was then repeated for 50 iterations to provide additional information about the distributions of the resulting statistics. The following is a description of the subsampling process.

Suppose the original 15-min time series data set is given by the formula

1 $${{{\boldsymbol q}_y}} {\rm{ = }}\left[ {{q_y}\left( 1 \right),{q_y}\left( 2 \right), \ldots {q_y}\left( r \right)} \right],$$

where ***q***_***y***_ is a vector (i.e., one dimensional matrix) containing records of flow rate for gauging station *y* from records 1 to *r*; *r* is the total number of records in the 15-min time series for each station. Now suppose that we randomly sample from ***q***_***y***_ based on the formula

2 $${\boldsymbol {qs}}{{\boldsymbol{s}}_{{\boldsymbol{y}},{\boldsymbol{i}}}}{\rm{ = }}\left[ {{q_y}\left( {{\rm{rs}}{{\rm{s}}_{y,i}}\left[ 0 \right]} \right),{q_y}\left( {{\rm{rs}}{{\rm{s}}_{y,i}}\left[ 1 \right]} \right), \ldots {q_y}\left( {{\rm{rs}}{{\rm{s}}_{y,i}}\left[ N \right]} \right)} \right],$$

where ***qss***_*y,i*_ is the subsample flow vector containing all subsampled records for gauging station *y* and iteration *i*. Because we require the subsample to be a random selection with on average even spacing between subsamples, we define the records that should be used for the subsamples used to develop ***qss***_*y,i*_ with the formula

3 $${\boldsymbol{rs}}{{\boldsymbol{s}}_{y,i}}{\rm{ = }}\left[ {\frac{S}{2} + n{\rm{*}}S + {\rm{R}}{{\rm{I}}_n}} \right]_{n = 0}^N,$$

where ***rss***_*y,****i***_ is the subsample record vector containing the randomly selected records used to develop the subsample flow vector ***qss***_*y,i*_. *S* is the average subsample interval (an even integer) and *n* is the subsample number ranging from 0 to *N*. The value of *N* is given by the formula

4 $$N{\rm{ = int}}\left( {{\rm{floor}}\left( {\frac{r}{S}} \right)} \right) - 1.$$

The functions int() and floor() select the nearest integer below *r*/*S*. For example, if *r*/*S* was 83.94, then the combined functions would return 83. RI_*n*_ is a random integer ranging from −*S*/2 to *S*/2. Offsetting $\frac{S}{2} + n{\rm{*}}S$ by RI_*n*_ ensures that each subsample will be somewhere within the range of *S* centered about $\frac{S}{2} + n{\rm{*}}S$. In our case, *S* was set to 96 (daily), 288 (three days), 672 (weekly), and 2922 (monthly). Per the minimum recommended number of bootstrap samples by Efron and Tibshirani (1993), 50 iterations (i) of ***rss***_*y,i*_ were developed for each gauging station (*y*) to assess the resulting distributions for minimum flow, maximum flow, and runoff volume.

To summarize the subsampling process: first, we developed subsample record vectors using Eq. 3 for each gauging station and iteration, and second, we developed subsample flow vectors using Eq. 2. In total, we developed 2500 subsamples (i.e., *y* sites times *i* subsamples, or 50 times 50) for each of the four subsample intervals S, for a total of 10,000 subsamples. The average size of each resulting subsample was 2571, 857, 367, and 84 records for daily, three day, weekly, and monthly subsampling intervals, respectively. A sample result of the subsampling procedure is presented in section Example subsampled hydrographs for the Truckee River near Farad (SiteID 10346000).

### Comparison Statistics

We compiled the 50 original 15-min data sets and the 10,000 subsamples into Microsoft Access SQL databases. SQL queries were developed to compute normalized statistical comparisons (see section Flow ratios) for the 15-min records and subsampled data. In all cases, the flow ratios were aggregated over the entire 7-year period (period) from the beginning of 2008 to the end of 2014. For purposes of comparison and normalization, the actual period minimum flow, maximum flow, and runoff volume for each station was determined from the 15-min data. As previously stated, each individual subsample observation was assumed to have the same flow measurement accuracy as the original 15-min observations.

#### Flow ratios

A normalized minimum flow ratio between minimum flow obtained from subsampled data for each gauging station (*y*) and iteration (*i*) (i.e., Qmin_*y,i*_) and actual minimum flow from 15-min record (i.e., Qmin_*a*_) expressed as a fraction (i.e., Qmin_*a*_/Qmin_*y,i*_) was used for comparison purposes. The actual minimum is placed in the numerator so that the minimum flow ratio ranges from 0 to 1.

A normalized maximum flow ratio between maximum flow obtained from subsampled data for each gauging station (*y*) and iteration (*i*) (i.e., Qmax_*y,i*_) and actual maximum flow from 15-min record (i.e., Qmax_*a*_) expressed as a fraction (i.e., Qmax_*y,i*_/Qmax_*a*_) was used for comparison purposes. Maximum flow ratio ranges from 0 to 1.

A normalized runoff ratio between runoff calculated from subsampled data for each gauging station (*y*) and iteration (*i*) (i.e., *V*_*y*,*i*_) and actual runoff from 15-min record (i.e., *V*_*a*_) expressed as a fraction (i.e., *V*_*y*,*i*_/*V*_*a*_) was used for comparison purposes. Runoff ratio ranges from 0 to infinity.

In all cases, if the denominator was 0, a value of 1 was returned. Ratios closer to 1 represent better agreement between subsampled data and the original 15-min records.

#### Correlation analysis

A correlation analysis was performed to assess relationships between minimum flow, maximum flow, and runoff ratios and the following variables: (1) latitude, (2) watershed area, (3) the R-B index, and (4) storage ratio. The first three variables were chosen to explore possible geographic, spatial scale, and temporal/magnitude-based dependencies, respectively. Storage ratio was selected because of the intuitive relationship between the “flattening” of the hydrograph discussed by Vörösmarty and Sahagian (2000) and the flow ratios being investigated. The results of the correlation analysis are presented in section Correlation analysis results. Note that there are mathematical dependencies between some variables; runoff ratio, R-B index, and storage ratio are each normalized by runoff (further discussed in section Correlation analysis results).

The R-B Index is a unitless value used to quantify the flashiness of a watershed (Baker et al. 2004). The R-B Index normalizes fluctuations in flow by the total flow over a given period, so that flashiness between watersheds can be compared. The entire 7-year study period was used for calculating the R-B Index.

Storage ratio is a unitless value calculated as the total usable reservoir water storage upstream of the gauging station divided by average annual runoff measured at the gauging station for the 7-year study period (Vörösmarty and Sahagian 2000). Usable reservoir water storage was calculated as the sum of the difference between maximum storage volume and dead pool volume for all reservoirs upstream of each gauging station. Storage potential of upstream soils, groundwater systems, and floodplains were not included in the storage ratio. The storage ratio attempts to normalize storage upstream of each gauging station, so that the impacts of reservoir storage can be quantitatively determined and compared among all gauging stations. Note that three storage ratios (SiteIDs 11051499, 11077500, and 11109800) are marked with an asterisk (*) in the supplemental materials. For these three sites, artificially imported water is stored in upstream reservoirs, so the amount of storage available is large compared to natural annual runoff. These three sites are not used in correlation analyses involving storage ratio.

### Hypotheses, Visualization Methods, and Evaluation Criteria

Table 1 provides a summary of the five visualization methods used in sections Flow ratio results and Correlation analysis results, organized by three hypotheses being evaluated. Criteria for evaluating each visualization method are provided in the right column.

**Table 1:** Summary of the three hypotheses and five visualization methods used in sections Flow ratio results and Correlation analysis results, along with evaluation criteria for each. Additional details for the third (3) hypothesis are presented in the text

Hypotheses	Visualization of results	Evaluation criteria
(1) Decreased observational frequency will negatively impact agreement between flow ratios computed from subsampled data and the continuous record	Tabular summary of sites with 50% of subsamples within ±10% and ±20% of actual flow ratios as a function of subsample interval (Table 2)	Closer to 50 indicates subsampled data better matches continuous records
(2) The nature of this impact will be different for each ratio	Quad box plots showing flow ratio distributions for all 50 sites for all subsample intervals (Figs. 3, 5, and 7)	Closer to 1 indicates subsampled data better matches continuous records
	Histograms of flow ratios showing site-subsample distributions organized by subsample interval (Figs. 4, 6, and 8)	Closer to 1 indicates subsampled data better matches continuous records
(3) There will be statistically significant correlations between the different flow ratios and latitude, watershed area, R-B Index, and storage ratio.*	Tabular summary of Pearson’s r values as a function of subsample interval (Table 3)	Farther from 0 (i.e., closer to 1 or −1) indicates stronger correlation
	Quad scatter plots of flow ratios as a function of latitude, watershed area, R-B Index, and storage ratio for daily subsample interval only (Figs. 10−12)	Visual interpretation for observable trends required

The following are additional sub-hypotheses related to the third (3) hypothesis in Table 1.

- Increasing latitude will improve estimates of maximum flow and runoff, but will worsen estimates of minimum flow.
- Increasing watershed area will improve estimates of minimum flow, maximum flow, and runoff.
- Increasing R-B Index will improve estimates of minimum flow, but will worsen estimates of maximum flow and runoff.
- Increasing storage ratio will improve estimates of minimum flow, maximum flow, and runoff.

## Results

### Example Subsampled Hydrographs

**Fig. 2:**
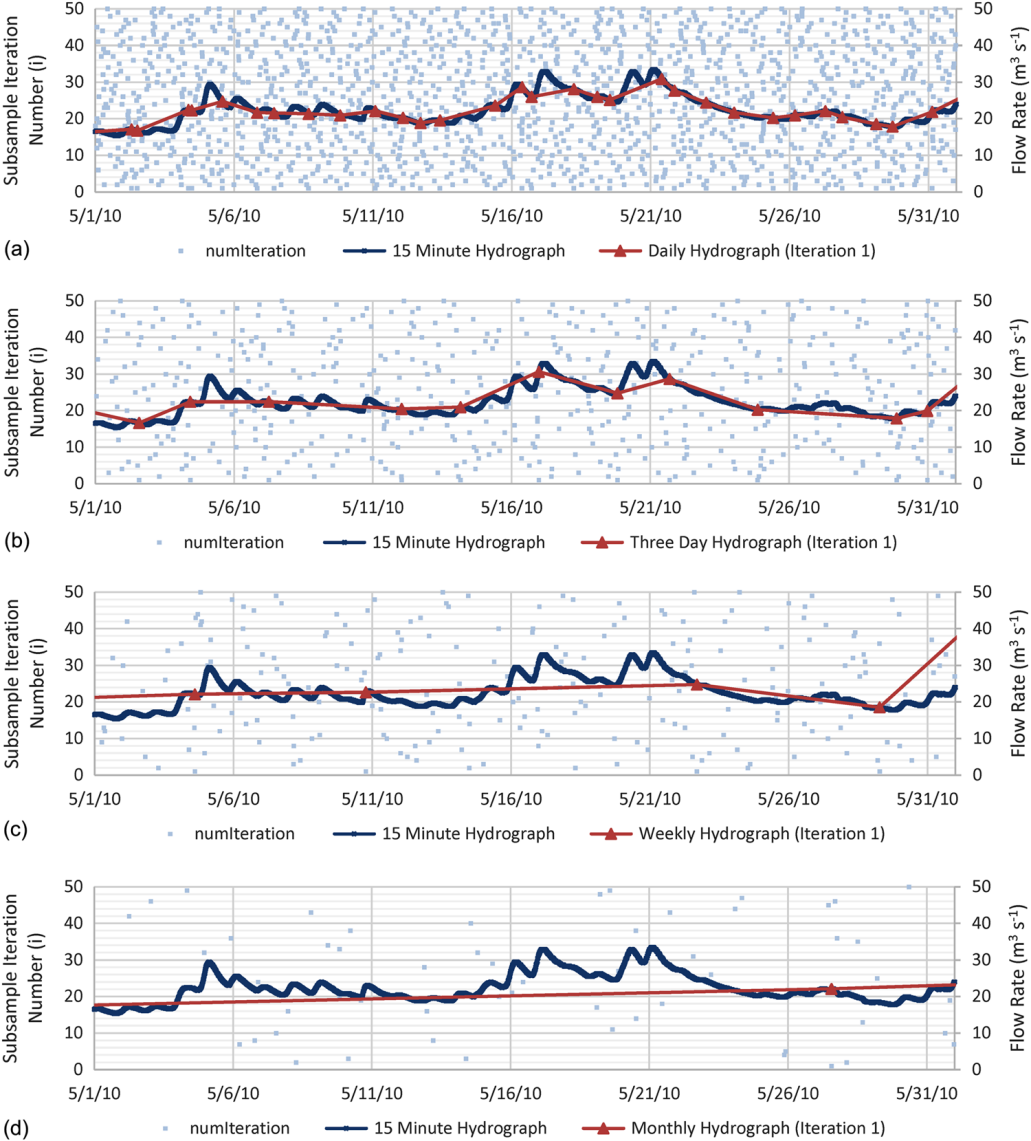
Example bootstrap randomized subsamples with replacement for each of the 50 iterations (*light blue dots*), original 15-min hydrograph (*dark blue*), and hydrograph from subsample iteration 1 (*red*) for the Truckee River near Farad. The *horizontal axis* displays time, in this case the month of May from 2010. The primary (*left*) *vertical axis* displays subsample iteration number (i), and the secondary (*right*) *vertical axis* displays flow rate. Each horizontal gridline represents a single subsample iteration from 1 to 50. Each *light blue square* on the horizontal gridlines represents a date time selected for the respective subsample iteration. The 30 subsamples that make up the hydrograph for iteration 1 shown as *red triangles* coincide horizontally, that is with respect to time, with the 30 *light blue squares* on the first horizontal gridline above the *x*-axis (i.e., subsample iteration number 1). The data shown are for **a** daily, **b** three day, **c** weekly, and **d** monthly subsample intervals

The subsampling selections and resulting hydrographs for daily, three day, weekly, and monthly subsample intervals are shown on Fig. 2 for the Truckee River near Farad (SiteID 10346000) near the California-Nevada state border for May 2010. Shown on each of the graphs (a–d) are (1) the original 15-min hydrograph (dark blue), (2) the subsampled hydrograph resulting from iteration 1 (red), and (3) the bootstrap randomized subsamples with replacement for each of the 50 iterations (light blue dots). The hydrograph represents a typical spring runoff superimposed with spring precipitation events in the Sierra Nevada mountains. The shorter scale temporal dynamics of the 15-min hydrograph are progressively lost as the subsample frequency decreases from daily to monthly. For example, the daily subsampled hydrograph shown by the red trace in Fig. 2a follows the general trends of the 15-min hydrograph shown in blue. However, the monthly subsampled hydrograph shown by the red trace in Fig. 2d almost completely misses the peaks and troughs shown in the 15-min hydrograph.

Each hydrograph can be constructed by (1) selecting a horizontal gridline representing a subsample iteration, and then (2) moving vertically from each light blue dot on the selected subsample iteration gridline until the 15-min hydrograph is reached. The random distribution of the roughly 1500, 500, 200, and 50 light blue dots, respectively, illustrates that the subsampling method described in section Subsampling procedure is providing good subsample randomization.

### Flow Ratio Results

Table 2 provides a summary of the number of sites that had at least half of the iterations of subsampled flow ratios within ±10 and ±20% of actual flow ratios for the four subsample intervals evaluated.

**Table 2:** Summary of the number of sites out of the 50 selected with at least half of the iterations (i.e., between the first and third quartile) with subsampled flow ratios within ±10 and ±20% of actual flow ratios for the four subsample intervals evaluated

Ratio	Subsample interval (S)	Daily	Three days	Weekly	Monthly
Min flow ratio	Number of sites within ±10%	39	35	31	23
	Number of sites within ±20%	42	38	36	25
Max flow ratio	Number of sites within ±10%	7	5	3	0
	Number of sites within ±20%	7	6	6	2
Runoff ratio	Number of sites within ±10%	44	39	27	12
	Number of sites within ±20%	49	46	43	22

For minimum flow ratio with a daily subsample interval, 39 and 42 of the 50 sites had a 50% chance that subsampled minimum flows were within ±10 and ±20% of the actual minimum, respectively. For the monthly subsample interval, 23 and 25 of the 50 sites had a 50% chance that subsampled minimum flows were within ±10 and ±20% of the actual minimum, respectively.

For maximum flow ratio with a daily subsample interval, only seven of the 50 sites had subsampled maximum flow within ±10 and ±20% of the actual maximum. None of the 50 sites had monthly subsampled maximum flows within ±10%, and only two were within ±20% of actual maximum flows.

For runoff ratio with a daily subsample interval, 44 and 49 of the 50 sites had a 50% chance that subsampled minimum flows were within ±10 and ±20% of the actual runoff, respectively. For the monthly subsample interval, 12 and 22 of the 50 sites had a 50% chance that subsampled runoff values were within ±10 and ±20% of actual runoff, respectively.

#### Minimum flow results

**Fig. 3:**
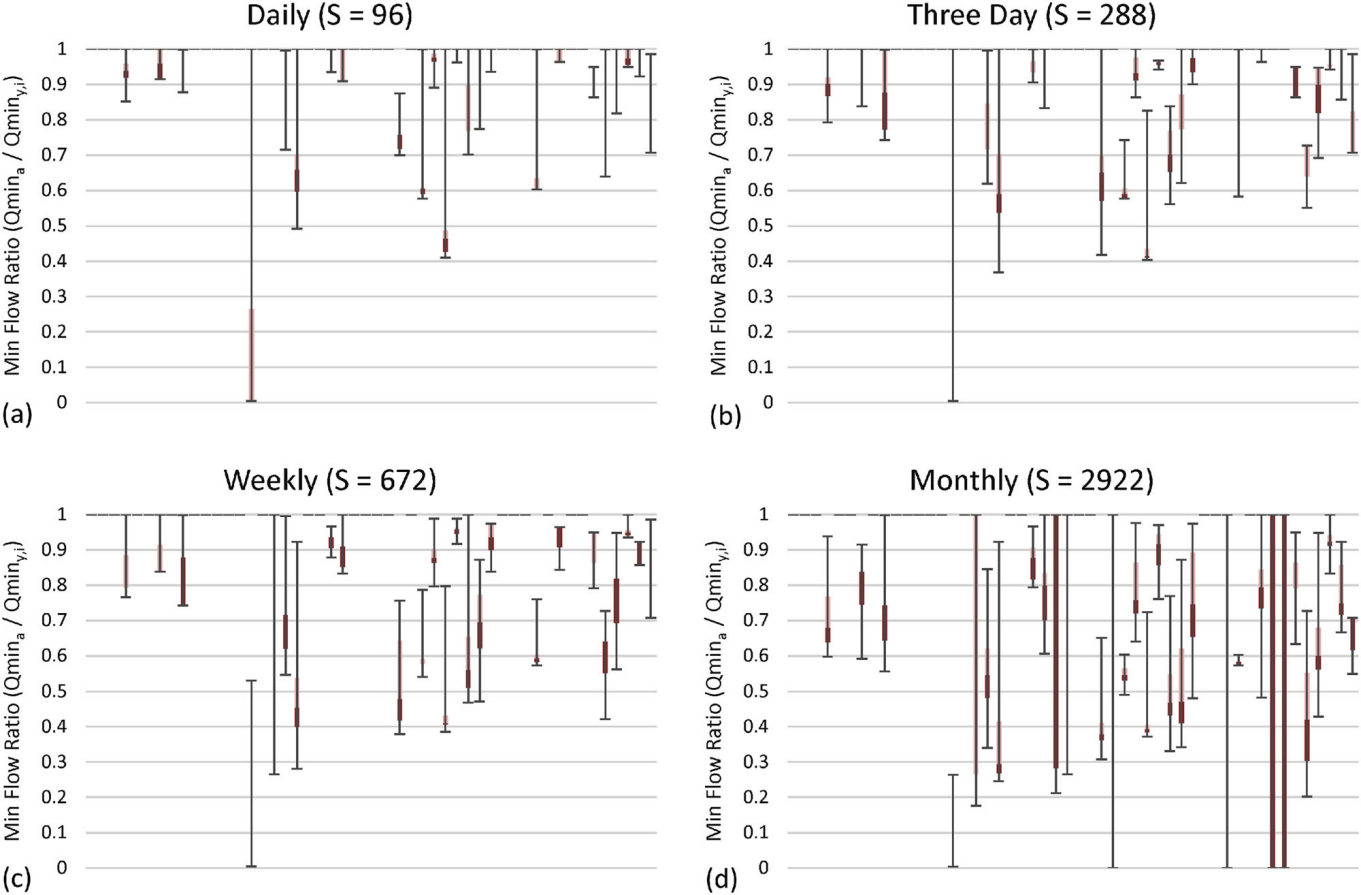
Minimum flow ratio between actual minimum flow from the 15-min record (i.e., Qmin_*a*_) and minimum flow calculated from subsampled data for each gauging station (*y*) and iteration (i) (i.e., Qmin_*y,i*_) and expressed as a fraction (i.e., Qmin_*a*_/Qmin_*y,i*_). All minimum flow ratios were calculated for the 7-year period from 2008 to 2014. Data for all four subsample intervals are shown starting from the *top left*. Each plot contains the median (Q2; interface between *light* and *dark red*), the 1st and 3rd quartiles (Q1 and Q3; bottom of *dark red* and top of *light red* respectively), and the minimum and maximum (negative and positive *error bars*, respectively). Note that sites with minimum, Q1, Q2, Q3, and maximum flow ratios equal to 1 are simply shown as a dash at the top of each graph. In cases where either Q1 and Q2, or Q2 and Q3 are coincident, no *light* or *dark red rectangles* are visible, respectively. Sites are sorted in ascending order by SiteID. The data shown are for **a** daily, **b** three day, **c** weekly, and **d** monthly subsample intervals

**Fig. 4:**
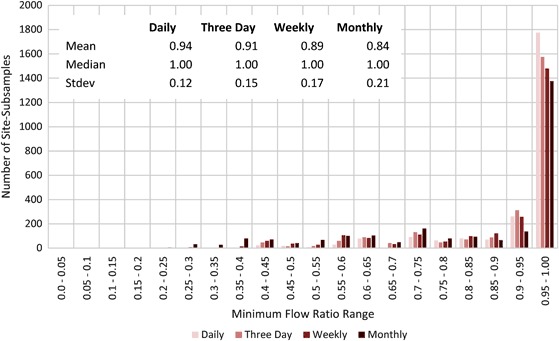
Histogram and basic statistics of minimum flow ratios for all four subsample intervals

Results for minimum flows are shown in Figs 3 and 4. The distribution of minimum flow ratios, shown as box plots in Fig. 3, moves progressively towards zero on the vertical axis as the subsample frequency decreases. Notice that the median (interface between light and dark red) minimum flow ratios moved progressively towards zero as the subsample frequency decreased. The closer the points are to 1 on the vertical axis, the better the subsampled data characterizes minimum flows.

A histogram of minimum flow ratios for daily, three day, weekly, and monthly subsample intervals (Fig. 4) shows non-normal distributions for all subsample intervals. The distributions for all subsample intervals were similar and were more heavily weighted towards the right, but increasingly less so as the subsample interval increased. Nearly 72% of the site-subsample pairs (site-subsamples) had a minimum flow ratio greater than or equal to 0.9.

#### Maximum flow results

**Fig. 5:**
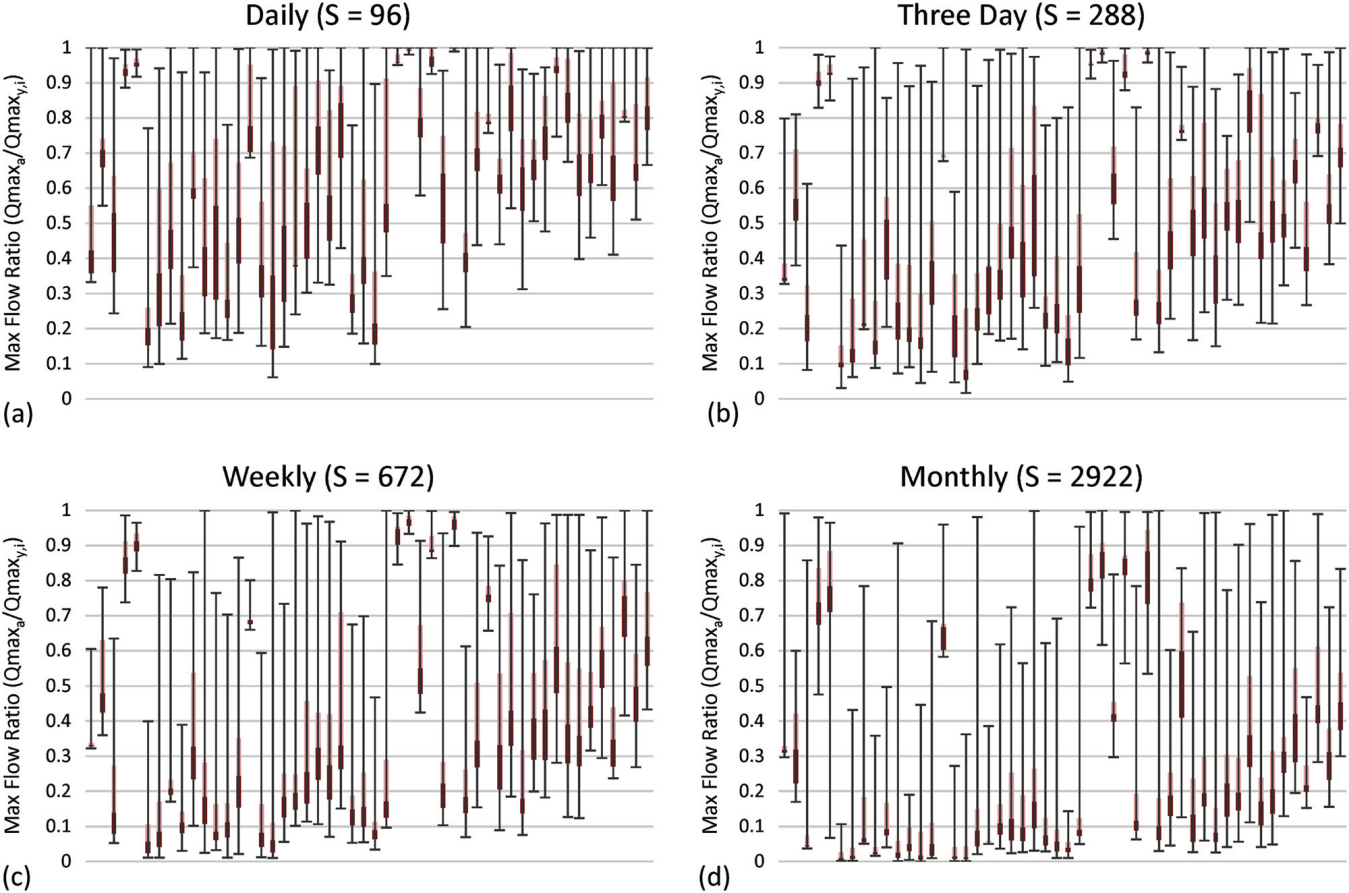
Maximum flow ratio between maximum flow calculated from subsampled data for each gauging station (*y*) and iteration (*i*) (i.e., Qmax_*y,i*_) and actual maximum flow from the 15-min record (i.e., Qmax_*a*_) expressed as a fraction (i.e., Qmax_*y,i*_/Qmax_*a*_). All maximum flow ratios were calculated for the 7-year period from 2008 to 2014. Data for all four subsample intervals are shown. Each plot contains the median (Q2; interface between *light red* and *dark red*), the 1st and 3rd quartiles (Q1 and Q3; bottom of *dark red* and top of *light red* respectively), and the minimum and maximum (negative and positive *error bars*, respectively). In cases where either Q1 and Q2, or Q2 and Q3 are coincident, no *light* or *dark red rectangles* are visible, respectively. Sites are sorted in ascending order by SiteID. The data shown are for **a** daily, **b** three day, **c** weekly, and **d** monthly subsample intervals

**Fig. 6:**
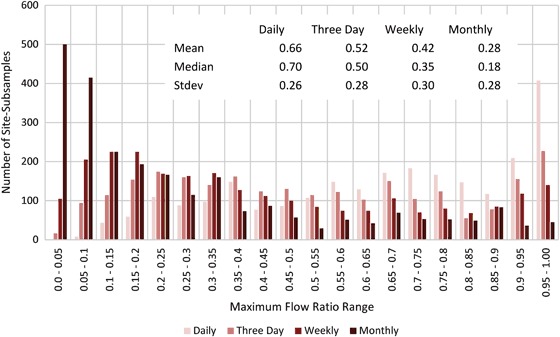
Histogram and basic statistics of maximum flow ratios for all four subsample intervals

Results for maximum flows are shown in Figs 5 and 6. Figure 5 shows box plots of the maximum flow ratios. The closer the points are to 1 on the vertical axis, the better the maximum flow was characterized. The median (interface between light and dark red), along with the distribution, moved progressively closer to 0 as the subsample frequency decreased. Even with a daily subsample interval, the median maximum flow ratios still ranged between 0.2 and 1.0, with an average of 0.67. This suggests that maximum flows were substantially underestimated, even with daily observations.

Figure 6 shows a histogram of maximum flow ratios for daily, three day, weekly, and monthly subsample intervals. The distributions for all subsample intervals were non-normal. The daily subsample distribution was more heavily weighted to the right, with 0.9 to 0.95 and 0.95 to 1.00 containing the highest number of site-subsamples (*n* = 617 or roughly 25%). In contrast, the monthly subsample distribution was more heavily weighted to the left, with 0.0 to 0.05, and 0.05 to 0.1 containing the highest number of site-subsamples (*n* = 915 or roughly 37%).

#### Runoff results

**Fig. 7:**
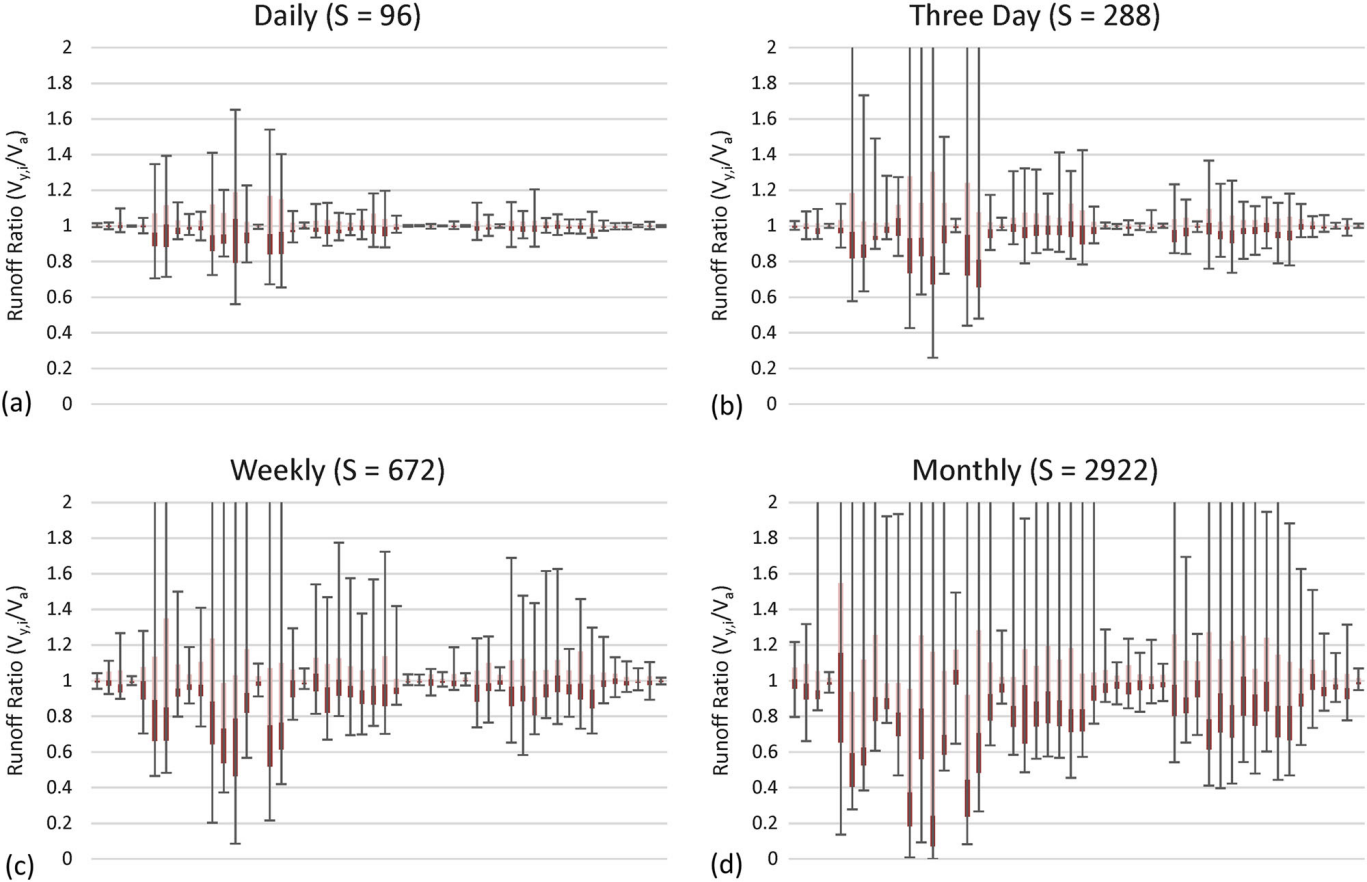
Runoff ratio between runoff calculated from subsampled data for each gauging station (*y*) and iteration (*i*) (i.e., V_*y,i*_) and actual runoff from 15-min record (i.e., V_*a*_) expressed as a fraction (i.e., V_*y,i*_/V_*a*_). Both runoff values are calculated for the 7-year period from 2008 to 2014. Data for all four subsample intervals are shown. Each plot contains the median (Q2; interface between *light* and *dark red*), the 1st and 3rd quartiles (Q1 and Q3; bottom of *dark red* and top of *light red*, respectively), and the minimum and maximum (negative and positive error bars respectively). Sites are sorted in ascending order by SiteID. The data shown are for **a** daily, **b** three day, **c** weekly, and **d** monthly subsample intervals

**Fig. 8:**
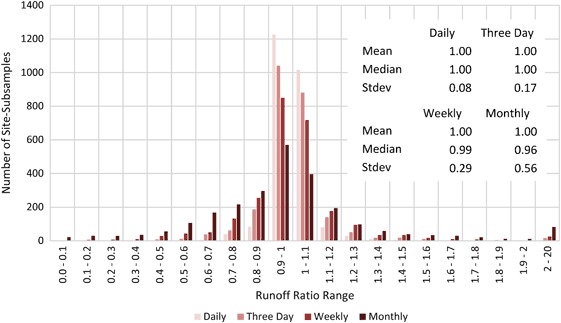
Histogram and basic statistics of runoff ratios for all four subsample intervals

Results for the runoff (volume) are shown in Figs 7 and 8. Figure 7 shows box plots of runoff ratios. The vertical axis scale is locked from 0 to 2, however, for subsample intervals greater than daily, some of the maximum runoff ratios (maximum error bars) were above 2 and are therefore not shown on the plot. The data move progressively farther from 1 as the subsample frequency decreases, indicating that runoff volume estimates became more uncertain as observation frequency decreased. The median values (interface between light and dark red) moved increasingly downwards from 1 as the subsample frequency decreased, representing an amplified negative bias in runoff estimates.

There was a systematic negative bias in the runoff estimates, as evidenced by the greater number of sites below 1 than above 1 for all subsample intervals. Runoff was underestimated for 54, 54, 55, and 61% of site-subsamples for daily, three day, weekly, and monthly subsample intervals, respectively. This indicates that the negative bias was stronger as the subsample frequency decreased. This trend is also illustrated by the median being consistently below the 1 runoff ratio line in Fig. 8, especially as the subsample frequency decreased to weekly and monthly.

**Fig. 9:**
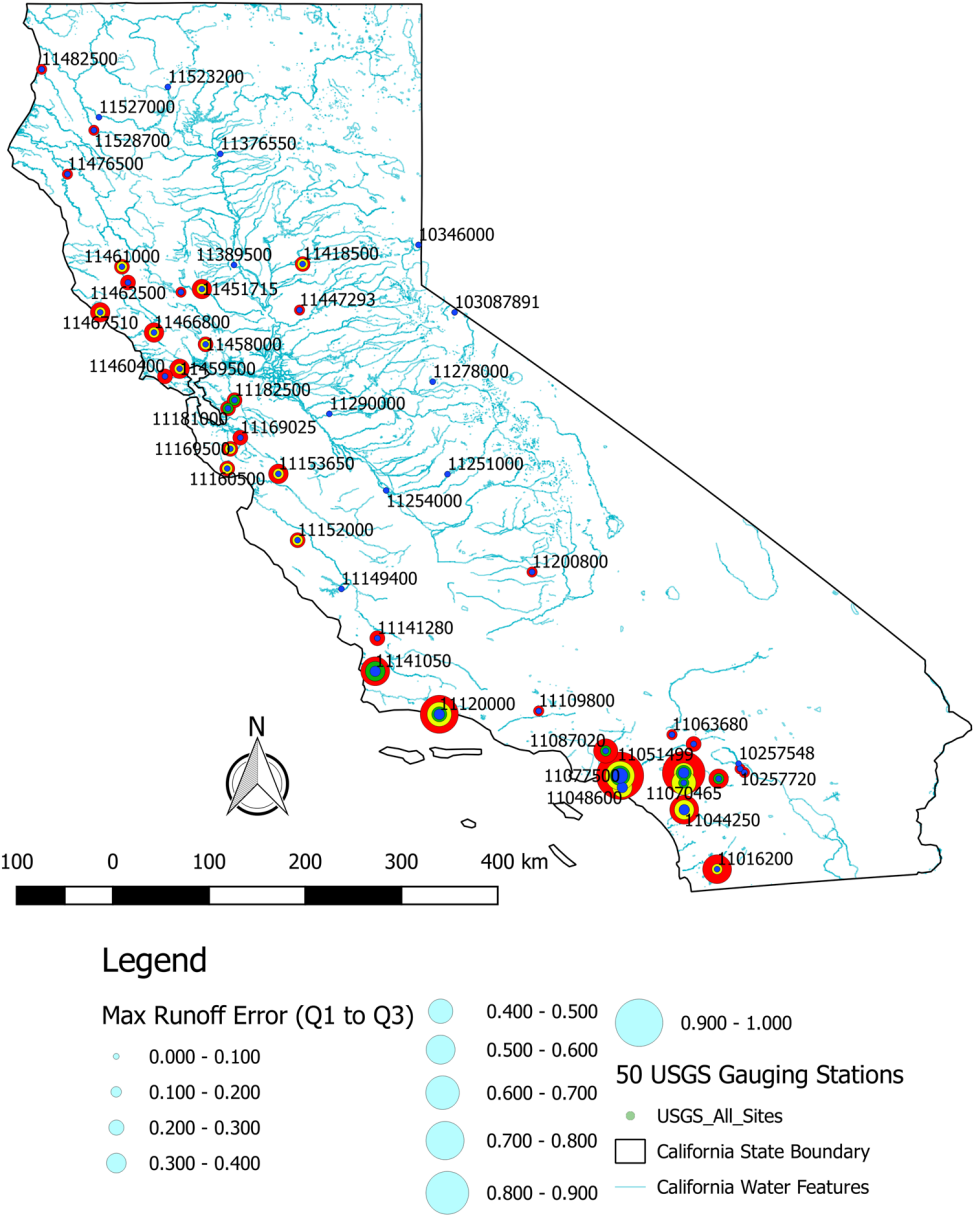
Map figure of 50 USGS stream gauges labeled with USGS Station ID. At each location, there are four concentric and scaled *circles*. The *circles* are scaled by maximum runoff error (i.e., maximum of the runoff ratio residuals) within the 1st and 3rd quartiles from the 50 subsample iterations. Daily, three day, weekly, and monthly subsample intervals are shown in blue, green, yellow, and red, respectively

Figure 9 presents a geographic summary of the subsampling results for runoff. At each location there are four concentric and scaled circles. Daily, three day, weekly, and monthly subsample intervals are shown in blue, green, yellow, and red, respectively. The size of the circle corresponds to the maximum from all 50 iterations of the absolute value of the runoff ratio minus one for the 1st and 3rd quartiles. In other words, there is a 50% chance that a runoff estimate would be within the displayed fraction of the actual runoff. For example, daily and monthly subsamples for Atascadero Creek near Goleta (SiteID 11120000) have a 50% chance of having runoff estimates within 16.8% (i.e., 0.168) and 76.4% (i.e., 0.764) of actual runoff, respectively. In general, watersheds in the San Francisco Bay Area (e.g., SiteIDs 11182500 and 11181000) and watersheds in Southern California (e.g., SiteIDs 11077500, 11070270, and 11070465) had the highest runoff ratio residuals for all subsample intervals. These watersheds also tend to exhibit greater flashiness, as indicated by higher R-B Index values.

### Correlation Analysis Results

**Fig. 10:**
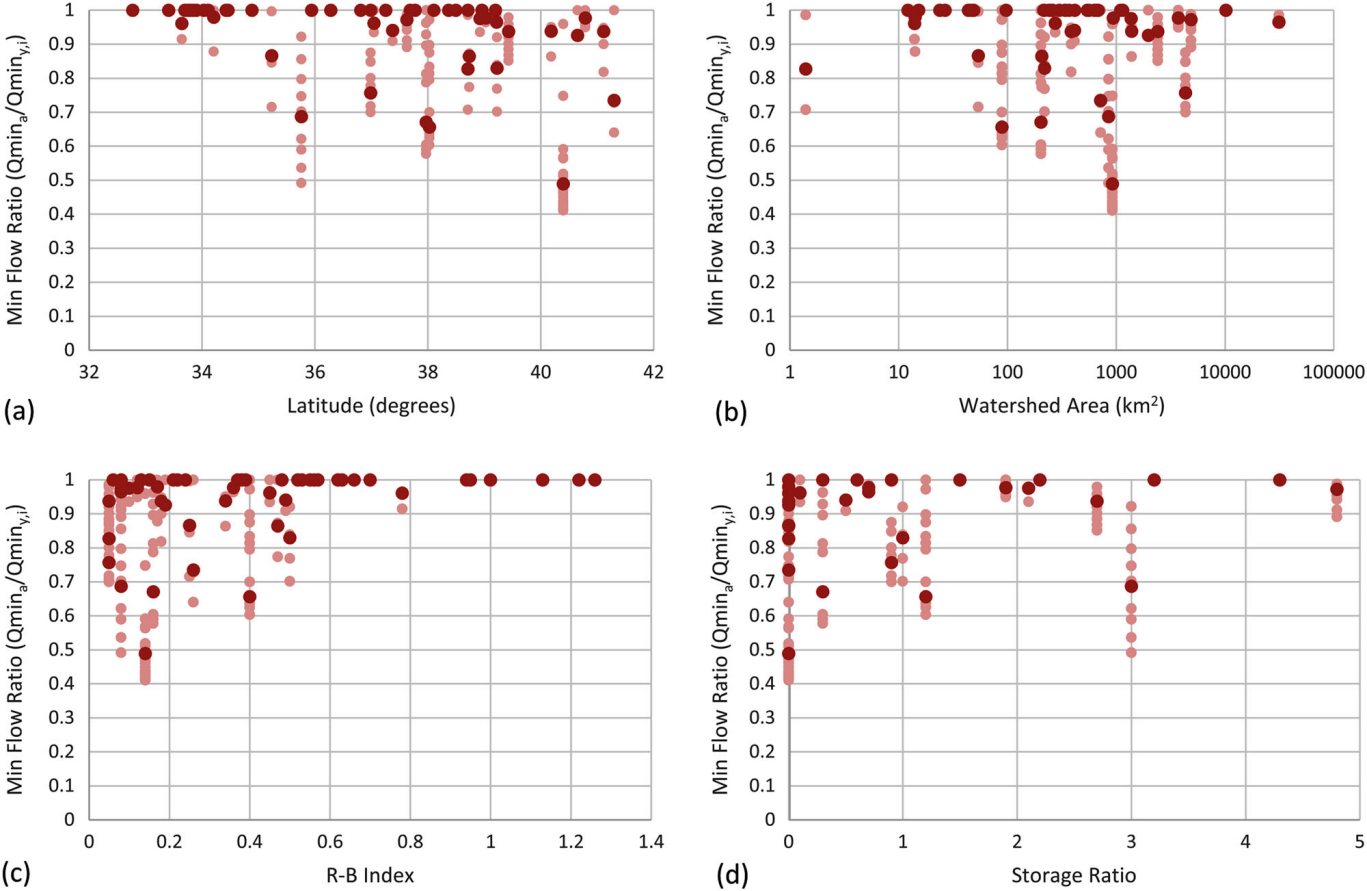
Scatter plots of minimum flow ratio as a function of **a** Latitude (decimal degrees), **b** watershed area (km^2^), **c** Richards-Baker flashiness index (R-B Index), and **d** storage ratio. Average data for all 50 sites shown in *dark red*; data for all 50 iterations shown in *light red*. Storage ratio calculated as the upstream usable reservoir storage divided by the mean annual runoff for the study period. Data shown for daily subsample interval only

**Fig. 11:**
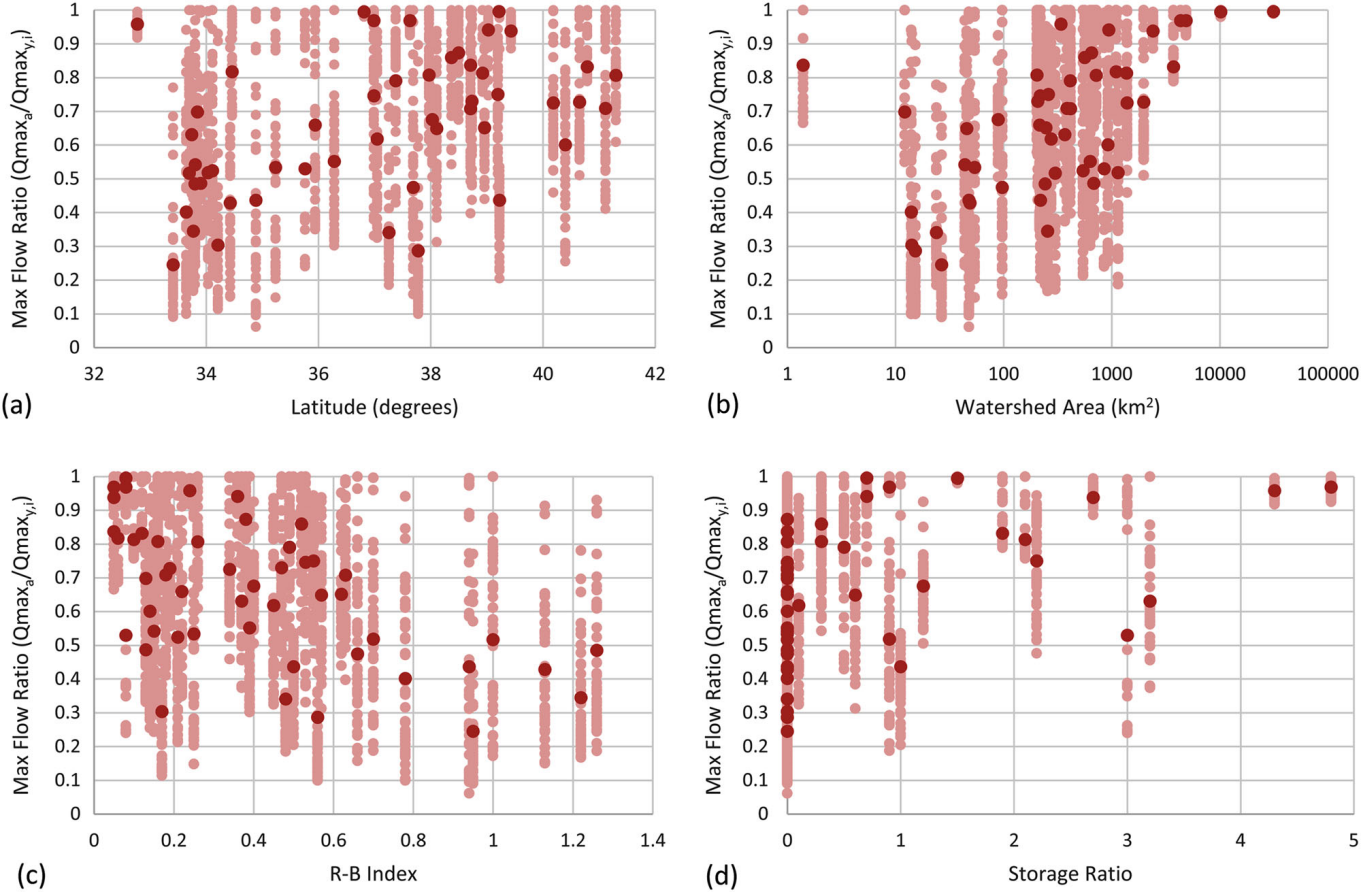
Scatter plots of maximum flow ratio as a function of **a** latitude (decimal degrees), **b** watershed area (km^2^), **c** Richards-Baker flashiness index (R-B index), and **d** storage ratio. Average data for all 50 sites shown in *dark red*; data for all 50 iterations shown in *light red*. Storage ratio calculated as the upstream usable reservoir storage divided by the mean annual runoff for the study period. Data shown for daily subsample interval only

**Fig. 12:**
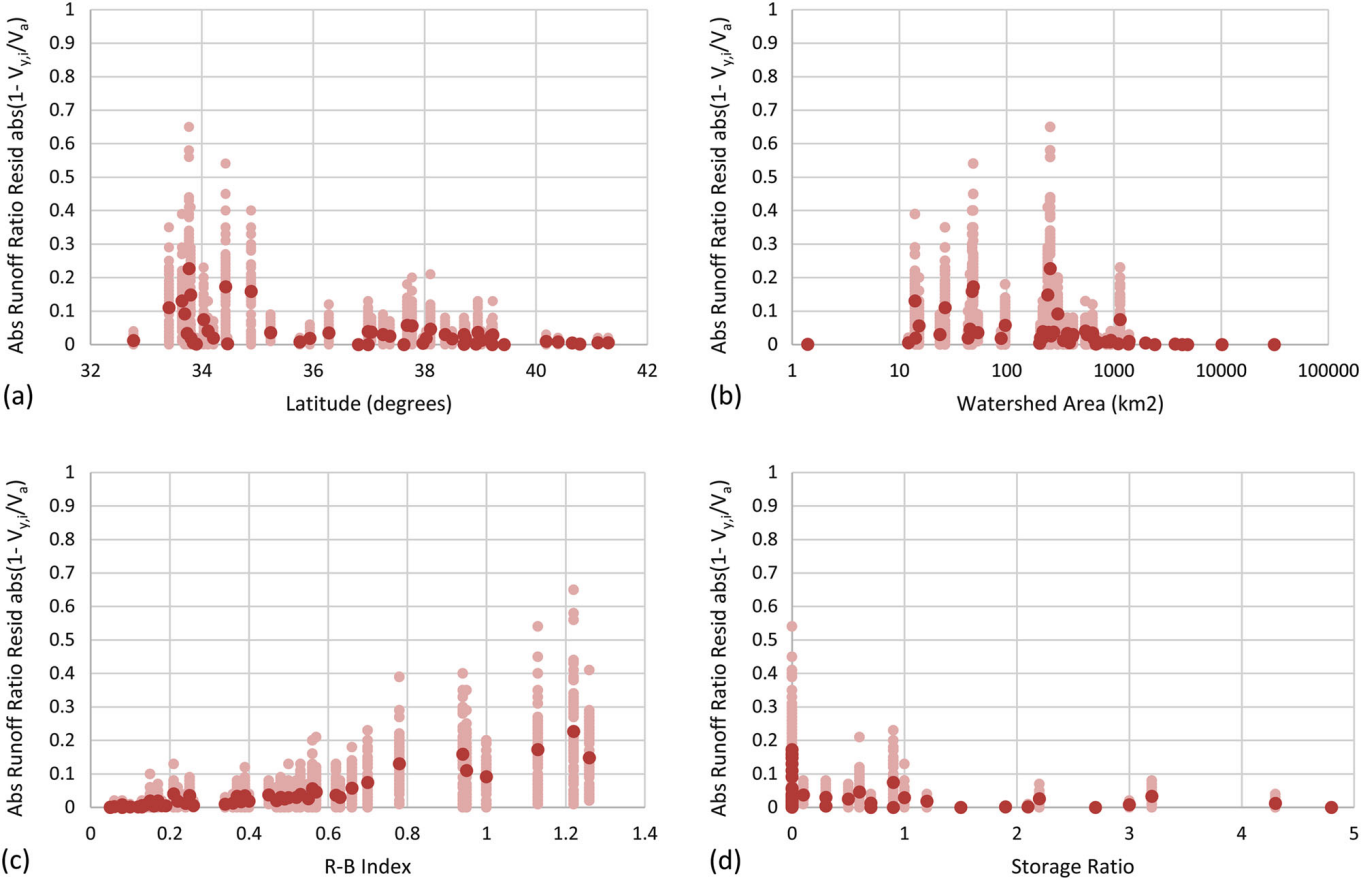
Scatter plots of the absolute value of the runoff ratio residual (i.e., 1−*V*_*y,i*_/*V*_*a*_) as a function of **a** latitude (decimal degrees), **b** watershed area (km^2^), **c** Richards-Baker flashiness index (R-B Index), and **d** storage ratio. Average data for all 50 sites shown in *dark red*; data for all 50 iterations shown in *light red*. Storage ratio calculated as the upstream usable reservoir storage divided by the mean annual runoff for the study period. Data shown for daily subsample interval only

Figures 10 to 12 show scatter plots between minimum flow, maximum flow, and runoff ratios, and (a) latitude, (b) watershed area, (c) R-B Index, and (d) storage ratio, respectively. Data are shown for daily sampling frequencies only. The dark red points are average values for each of the 50 sites. The light red points show the 50 iterations for each of the 50 sites. Table 3 shows Pearson’s *r* values between average flow ratios (i.e., one value per site; total of 50) and (1) latitude, (2) watershed area, (3) R-B Index, and (4) storage ratio. Pearson’s *r* values were tested for significance with a two-tailed *p*-value hypothesis test (*n* = 50, *p* = 0.05; Table 3); statistically significant values are shown with bold and italic font (i.e., Pearson’s *r* > 0.28). Values shown in dark red had mathematical dependencies between variables (see note under Table 3); therefore, significance tests are non-valid, so values have regular font styles.

**Table 3:** Pearson’s *r* values (i.e., correlation coefficients) between average flow ratios and (1) latitude (decimal degrees), (2) watershed area (km^2^), (3) R-B index (unitless), and (4) storage ratio (unitless)

Data used from all 50 sites for all four subsample intervals. Statistically significant (two tailed; *p* = 0.05) Pearson’s *r* values shown in bold italic font

^a^ Values shown in dark red have mathematical dependencies between variables; Runoff Ratio, R-B Index, and Storage ratio are each normalized by runoff. Therefore, dark red values cannot be compared to light red values, but can be compared in a relative sense to other dark red values. Note that statistical significance is also impacted by this dependency

There were statistically significant correlations between subsampled average minimum flow ratios and latitude and R-B Index; no significant correlations were seen with watershed area and Storage Ratio (Table 3 and Fig. 10). In general, this indicated that minimum flow estimates became more accurate as latitude decreased and as flashiness increased. The strength of the statistically significant correlations increased as subsample frequency decreased.

There were statistically significant correlations between subsampled average maximum flow ratios and latitude, watershed area, R-B Index, and storage ratio (Table 3 and Fig. 11). In general, this indicated that maximum flow estimates became more accurate as latitude, watershed area, and storage ratio increased, and R-B index decreased. The strength of the watershed area, R-B index and storage ratio correlations increased as subsample frequency decreased. In contrast, the strength of the correlation with latitude decreased as subsample frequency decreased.

There were statistically significant correlations between subsampled average runoff ratio and latitude (Table 3 and Fig. 12; see note below Table 3). In general, this indicated that runoff estimates became more accurate as latitude increased. The strength of this correlations was relatively unaffected by decreased subsample frequency.

## Discussion

Accurate streamflow statistics of minimum flow, maximum flow, and runoff often form the basis of sound water resource management and planning. Assuming (1) subsampled water level observations are as precise and accurate as continuous observations and (2) an equally accurate stage-discharge curve is available for converting observed water levels to flows, this analysis indicates that lower frequency observations of stream stage and flow can be useful, and could play a role in hydrologic data generation. The utility of lower frequency data depends largely on what the ultimate use(s) of the data are. Table 4 provides a summary of the discussion organized by the hypotheses presented in Table 1.

**Table 4:** Summary of discussion organized by hypotheses

Hypotheses	Summary of results (see sections Minimum flow, Maximum flow, and Runoff for details)
(1) Decreased observational frequency will negatively impact agreement between flow ratios computed from subsampled data and the continuous record	Decreased observational frequency negatively impacted minimum flow, maximum flow, and runoff ratios calculated from the subsampled data as compared to the original 15-min USGS record (Table 2 and Figs. 3–8)
(2) The nature of this impact will be different for each ratio	The nature of this impact was different depending on the flow ratio in question. Maximum flow ratio was least sensitive to changes in observational frequency, whereas runoff ratio was most sensitive, with minimum flow ratio being moderately sensitive (Table 2 and Figs. 3–8)
(3) There will be statistically significant correlations between the different flow ratios and latitude, watershed area, R-B Index, and storage ratio	There were statistically significant correlations between some average flow ratios and latitude, watershed area, R-B Index, and storage ratio, with R-B Index having the most significant correlation with maximum flow (Table 3 and Figs. 10–12)

One limitation of our approach was the assumption that citizen science spot measurements of water level or stage could be converted to flow with the same accuracy as 15-min continuous USGS records. Much of the challenge of streamflow monitoring lies precisely in the conversion from stage to flow, or the development of the stage-discharge rating curve (Braca 2008). For example, many of the USGS rating curves implicitly utilized in this analysis were developed by trained hydrometric professionals using sophisticated and expensive equipment over the course of several decades. In addition to uncertainties in water level observations, the discussion about Citizen Hydrology should also focus on understanding uncertainties in rating curves (Mason et al. 2016; McMillan and Westerberg 2015; Domeneghetti et al. 2012 and others), focusing on those developed from infrequent observations, or on new methods for Citizen Hydrologists to accurately observe streamflow directly. The associated uncertainties with these new methods will need to be assessed to capture the comprehensive uncertainties of Citizen Hydrology data.

### Minimum Flow

Estimates of minimum flow discussed in section Minimum flow results, as compared to maximum flow and runoff (sections Maximum flow results and Runoff results, respectively), were the least sensitive to changes in subsample intervals. Because minimum flows tend to persist for longer timescales, they were estimated within 10% for half of the subsample iterations at 39 (daily) and 23 (monthly) of the 50 sites. There were statistically significant correlations between subsampled average minimum flow ratios and latitude and R-B Index. Precipitation in California has a positive correlation with latitude. We suggest that the observed negative correlation between latitude was due to north-to-south trends in precipitation, resulting in fewer ephemeral streams and more variable minimum flows as latitude increases. Subsampled measurements are most likely to characterize minimum flows for ephemeral streams, or streams that normally go dry for at least certain parts of the year. Streams that run dry also typically have a higher flashiness index.

### Maximum Flow

Because maximum flows occur only briefly, it is unlikely that reliable maximum flow estimates (section Maximum flow results) will be obtained from subsampled measurements with average observation intervals of daily or greater. For example, maximum flows were estimated within 10% for half of the subsample iterations at only 7 (daily) and 0 (monthly) sites. This is consistent with Cheviron et al. (2014) who found that only observation intervals that are smaller than the characteristic time period of fluctuations in the variable of interest tend to ensure reliable approximations. Therefore, if the primary monitoring objective is developing data for water resources infrastructure design, whereby maximum flows are required as design criteria, we suggested either (1) variable observation frequency based Citizen Hydrology (e.g., it is raining so go take measurements; see section Variable observation frequencies) or traditional continuous stream gauging methods. Our results also indicate that a simple mechanical maximum level gauge with a manual reset similar to that discussed by Bragg et al. (1994) could be an important addition to Citizen Hydrology flow monitoring sites if maximum water levels and flows need to be assessed. There were statistically significant correlations between subsampled maximum flow ratios and latitude, watershed area, R-B Index, and storage ratio (Table 3 and Fig. 11). The strongest correlations were between maximum flow ratios and R-B Index, followed closely by storage ratio and watershed area. One of the strongest controls on the timescales of the rainfall-runoff relationship is watershed area. All else being equal, larger watersheds have more temporally damped runoff responses, and vice versa. Additionally, significant reservoir water storage (i.e., high storage ratio) can drastically affect stream hydrographs, with one of the significant impacts being a “flattening” of the hydrograph (Vörösmarty and Sahagian 2000). This “flattening” of the hydrograph increases chances of characterizing maximum flows with lower frequency observations, especially as observation frequency decreases. Therefore, these results were congruent with our intuitions, and are similar to those discussed by Horowitz et al. (2015).

### Runoff

Runoff volumes were estimated within 10% for half of the iterations at 44 (daily) and 12 (monthly) of the 50 sites. The systematic negative bias in runoff estimates that increased as the subsample frequency decreased is congruent with the findings of Coynel et al. (2004). Data assimilation could be helpful to correct for these biases (see section Data assimilation). For daily observations on streams with average flows greater than 0.2 m^3^ s^−1^, or storage ratios greater than one, runoff was estimated within 20% (except for one site) and 10%, respectively for half of the subsample iterations. There were statistically significant correlations between subsampled runoff residuals and latitude and watershed area (Table 3 and Fig. 12). There are mathematical dependencies between runoff ratio and R-B Index and storage ratio, because each are normalized by runoff. Therefore, Pearson’s *r* for these relationships should not be directly compared to other Pearson’s *r* values. Additionally, statistical significance is also impacted by this dependency. Since runoff residuals closer to zero indicate more accurate characterizations of runoff, negative correlations with latitude, watershed area, and storage ratio suggest runoff estimates improve as these variables increase. Congruent with intuition, the positive correlation between runoff ratio and R-B Index indicates that runoff can be more accurately estimated from low frequency observations in watersheds with low flashiness (and vice versa). Short period runoff events in flashy ephemeral streams often contribute significant percentages of total runoff. It is more likely that lower frequency measurements will produce less accurate runoff results, because critical portions of the hydrograph can be completely missed as the observation frequency increases.

### Variable Observation Frequencies

While the subsampling procedure used in this paper produced somewhat regularly spaced readings, actual Citizen Hydrology observations will likely consist of an irregular mixture of observation frequencies. Thoughtfully varied observation frequencies, however, are a potential strength of Citizen Hydrology. We envision that, at a minimum, monitoring frequencies could be varied based on (1) typical seasonal hydrologic patterns and (2) individual rainfall-runoff events. In Nepal, for example, where our field work is being completed, it rains for roughly 4 months during the monsoon season (June–September), and is relatively dry for the remaining 8 months. Hydrographs during the monsoon season are quite dynamic, and therefore more frequent observations are desired. During the dry period, the hydrograph mainly undergoes a long recession, so less observations are needed, especially towards the end of the recession prior to the next monsoon. Additionally, depending on rainfall-runoff response timescales, observation frequencies could be altered depending on rainfall duration and intensity, or more simply by if it is raining or not. Therefore, future work should explore how variable observation frequencies, or adaptive monitoring, could lower uncertainty in Citizen Hydrology data.

### Data Assimilation

We suggest that data assimilation (briefly mentioned in section Runoff), or a systematic combination of modeling and observations, could be promising methodology for adding value to, and improving accuracy of, Citizen Hydrology observations. For example, higher frequency observations of rainfall collected by a permanently installed sensor could be combined with lower frequency observations of stream stage and flow performed by Citizen Hydrologists. Then, in the context of a rainfall-runoff model, these data could be combined to help “fill in the gaps” of the hydrograph. Data assimilation has the possibility to improve minimum flow, maximum flow, and runoff estimates based on lower frequency observations, and should be the focus of future Citizen Hydrology research.

### Relevance for Data Poor Regions

The results of this research are most meaningful if the watersheds chosen for subsampling from the “data rich” region(s) are similar to those of the “data poor” region(s) targeted for applications of Citizen Hydrology. For our purpose of designing a Citizen Hydrology monitoring campaign in Nepal, we specifically chose stream gauges from California for subsampling because of (1) the abundance of high quality stream gauging stations and (2) the topographic and climate similarities with Nepal. For example, both California and Nepal have well-defined 4 to 5 month long wet periods when the majority of precipitation occurs (i.e., November–March and June–September, respectively), followed by prolonged dry periods. During the wet periods, both California and Nepal have significant precipitation events that occur due to the strong winter Pacific jet stream (Dettinger et al. 2011) and the Asian Summer Monsoon (Ramage 1971), respectively. Additionally, both California and Nepal have significant topographic variations in a direction perpendicular to the predominant direction of the jet stream. In the case of California, low pressure systems from the Pacific Ocean typically move to the east, and are forced over the Sierra Nevada mountains, which predominantly run north to south. In Nepal, the South Asian monsoon moves to the north, while the Himalayas predominantly run east to west. While results from this analysis can be used to inform Citizen Hydrology efforts in “data poor” regions with dissimilar hydrologic contexts to that of California, it is suggested that the subsampling procedures discussed herein be repeated for hydrologically similar “data rich” regions.

As a sample “data poor” region application, we are using Citizen Hydrology observations to estimate runoff in several sub-watersheds (10–587 km^2^) of the Bagmati River watershed in the Kathmandu Valley. Precipitation patterns and amounts for the Kathmandu Valley are similar to those in Northern California (i.e., above a latitude of roughly 36 north). There are 31 watersheds with a latitude above 36 included in this study ranging in size from 1 to 31,313 km^2^. The highest R-B Index observed for these 31 sites was 0.66 for SiteID 11181000. For daily observation frequencies, out of a total of 1550 site-subsamples (i.e. 31 sites times 50 subsamples), only 28 site-subsamples had runoff errors greater than 10%, and only one site-subsample exceeded 20%; the average runoff error was 1.9%. With the assumptions previously stated at the end of the section Background and introduction in mind (i.e. regarding water level observation accuracy and stage-discharge curve availability), these results give us reasonable confidence that runoff estimates based on daily Citizen Hydrology observations should be within 10% of actual runoff, if not better.

## Summary and Conclusions

The goal of this paper was to investigate the impacts of lower frequency observations (i.e., daily, three day, weekly, and monthly), similar to those that could be produced by Citizen Hydrology, on the accuracy of basic streamflow statistics like minimum flow, maximum flow, and runoff. To answer this question, we performed a subsampling analysis on 7 years of streamflow data from 50 USGS gauging stations in California. Depending on the questions being asked, and the characteristics of the watershed(s) in question, lower frequency observations, such as those produced from Citizen Hydrology, can provide useful hydrologic information. In general, as watershed flashiness decreases and storage ratio increases, the reliability of minimum flow, maximum flow, and runoff estimates obtained from low frequency observations increases. Also, as latitude increases, which for California is a reasonable proxy for precipitation, the reliability of runoff estimates based on low frequency observations increases. Interestingly, watershed size seems to play a less prominent role than latitude (i.e., precipitation), R-B Index, and storage ratio in determining reliability of low frequency observation based runoff estimates.

## Electronic supplementary material

Supplementary Material

Supplementary Material

Supplementary Material

Supplementary Material
