# Supplementary figures and images for: Continuity vs. the Crowd—Tradeoffs Between Continuous and Intermittent Citizen Hydrology Streamflow Observations
Source: Environ Manage. 2017 Apr 25;60(1):12–29. doi: 10.1007/s00267-017-0872-x (PMC5486826; doi:10.1007/s00267-017-0872-x)

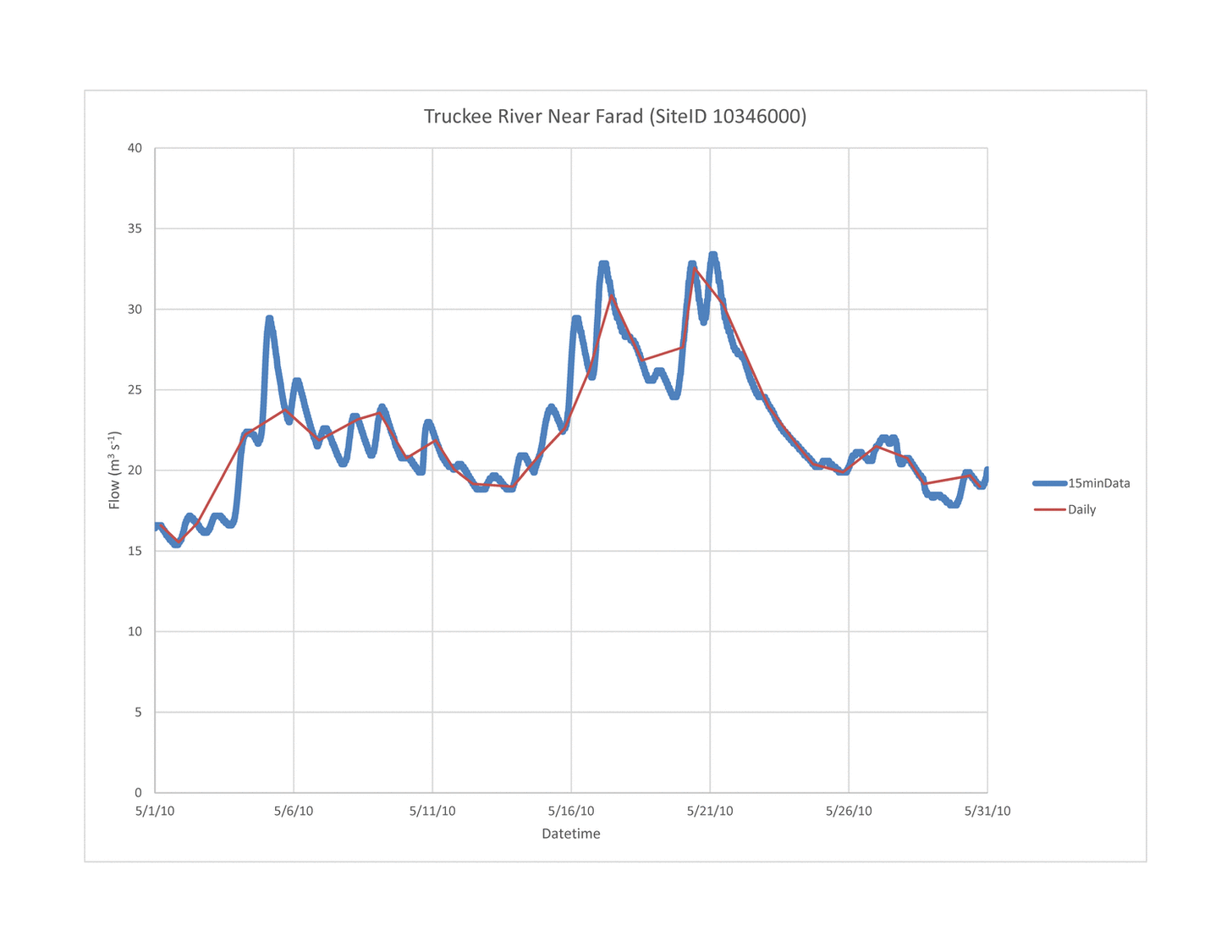

Supplement: Supplementary file 1 — Supplementary Material [file 267_2017_872_MOESM1_ESM.gif]

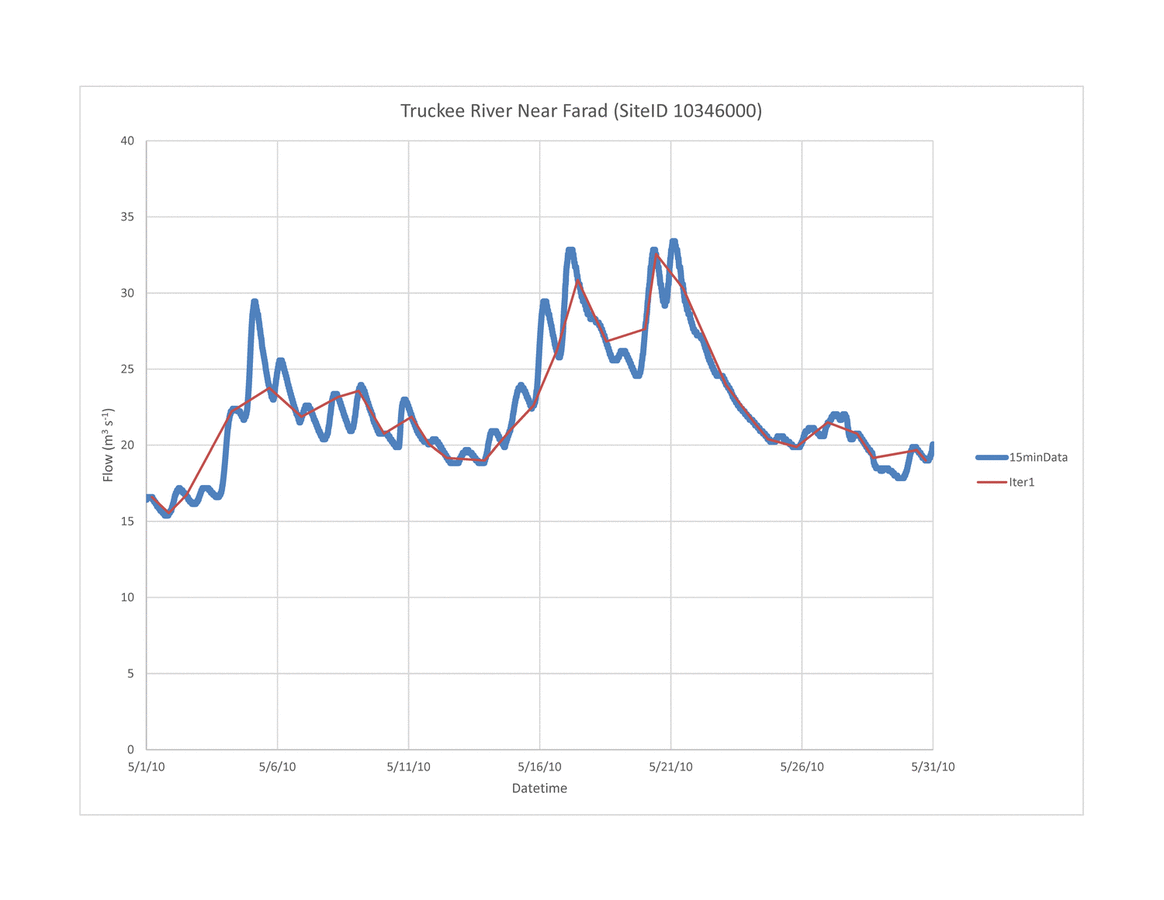

Supplement: Supplementary file 2 — Supplementary Material [file 267_2017_872_MOESM2_ESM.gif]
