# Supplementary material for: Continuity vs. the Crowd—Tradeoffs Between Continuous and Intermittent Citizen Hydrology Streamflow Observations
Source: Environ Manage. 2017 Apr 25;60(1):12–29. doi: 10.1007/s00267-017-0872-x (PMC5486826; doi:10.1007/s00267-017-0872-x)
Supplement: Supplementary file 4 — Supplementary Material [file 267_2017_872_MOESM4_ESM.pdf]

SmartPhones4Water (S4W)

S4W is a nonprofit 501(c)(3) organization that is passionate about (1) people in the margins and (2) wise stewardship of our natural resources. We believe that water links these themes in the most profound ways.

Water is our most precious resource. A famous Scottish mathematician, Lord Kelvin, once said, “you can’t manage a resource you don’t measure.”

The traditional approach to collecting water data (e.g. water levels in streams and groundwater, water quality, etc.) requires permanent sensors and is expensive, easily disrupted by corruption, is reliant on often weak political institutions, and simply doesn’t work in many cases.

S4W = crowdsourcing + mobile technology. S4W uses an Android application called Open Data Kit (ODK) to collect data about water with citizen scientists. Advances in mobile technology (e.g. GPS, cameras, etc.) have drastically improved the accuracy and reliability of citizen science observations. All data collecting by S4W is public domain.

S4W has a three pronged approach of Research, Education, and Employment. Our first pilot project is in the Kathmandu Valley of Nepal.

Our research partners include Delft University of Technology, Stockholm University, Chico State University, and NAMI University, Kathmandu Institute of Applied Technology, and CORAM-Nepal. Our research goals are to provide a proof of concept that will lead the way towards scaling this approach into the rest of Nepal and beyond.

S4W has a strong emphasis on educational training of our local citizen scientists. We have partnerships with over 30 secondary schools in the Kathmandu Valley, and have over 20 Nepali undergraduate students and over 10 foreign graduate student volunteers.

We currently employee roughly 10 citizen scientists, and have the goal of scaling this to over 100 by June 2017. By participating in the S4W-Nepal project these citizen scientists can supplement their normal income by an extra 5% to 30%, providing a key buffer between the poverty line.

The first step to solving any problem is thoughtfully characterizing it. Once the problem is characterized, a plan can be developed and then implemented. Many parts of the developing world

have huge issues with sustainable water management. S4W aims to generate the data that are necessary to characterize these issues, so that solutions can be developed, and ultimately implemented.

Ways To Get Involved

If you like what you are hearing and want to get involved, here are a few next steps:

- Like/Share our CrowdRise page with your social media networks (https://www.crowdrise.com/smartphones4water)
- Make a tax deductible donation via CrowdRise
    - If you want to make a donation larger than $100 you can send checks to the US address listed on our website
- Sign up for our mailing list (http://www.smartphones4water.org/category/news/)
- Hold a Phone Drive at Your Business - Collect old phones from employees/family/friends (we have an office in Chico that can send boxes to)
- Come to Nepal and check things out in person (free room and board if you come!)
